# Mapping integration of midwives across the United States: Impact on access, equity, and outcomes

**DOI:** 10.1371/journal.pone.0192523

**Published:** 2018-02-21

**Authors:** Saraswathi Vedam, Kathrin Stoll, Marian MacDorman, Eugene Declercq, Renee Cramer, Melissa Cheyney, Timothy Fisher, Emma Butt, Y. Tony Yang, Holly Powell Kennedy

**Affiliations:** 1 Birth Place Lab, Department of Family Practice, Faculty of Medicine, University of British Columbia, Vancouver, British Columbia, Canada; 2 University of Sydney, School of Medicine, Sydney, Australia; 3 Maryland Population Research Center, University of Maryland, College Park, Maryland, United States of America; 4 School of Public Health, Boston University, Boston, Massachusetts, United States of America; 5 Law, Politics and Society, Drake University, Des Moines, Iowa, United States of America; 6 Department of Anthropology, Oregon State University College of Liberal Arts, Corvallis, Oregon, United States of America; 7 Department of Obstetrics and Gynecology, Geisel School of Medicine, Dartmouth University, Lebanon, New Hampshire, United States of America; 8 Health Administration and Policy, George Mason University, Fairfax, Virginia, United States of America; 9 Department of Midwifery, Yale School of Nursing, Orange, Connecticut, United States of America; University of Rochester, UNITED STATES

## Abstract

Poor coordination of care across providers and birth settings has been associated with adverse maternal-newborn outcomes. Research suggests that integration of midwives into regional health systems is a key determinant of optimal maternal-newborn outcomes, yet, to date, the characteristics of an integrated system have not been described, nor linked to health disparities.

**Methods:**

Our multidisciplinary team examined published regulatory data to inform a 50-state database describing the environment for midwifery practice and interprofessional collaboration. Items (110) detailed differences across jurisdictions in scope of practice, autonomy, governance, and prescriptive authority; as well as restrictions that can affect patient safety, quality, and access to maternity providers across birth settings. A nationwide survey of state regulatory experts (n = 92) verified the ‘on the ground’ relevance, importance, and realities of local interpretation of these state laws. Using a modified Delphi process, we selected 50/110 key items to include in a weighted, composite Midwifery Integration Scoring (MISS) system. Higher scores indicate greater integration of midwives across all settings. We ranked states by MISS scores; and, using reliable indicators in the CDC-Vital Statistics Database, we calculated correlation coefficients between MISS scores and maternal-newborn outcomes by state, as well as state density of midwives and place of birth. We conducted hierarchical linear regression analysis to control for confounding effects of race.

**Results:**

MISS scores ranged from lowest at 17 (North Carolina) to highest at 61 (Washington), out of 100 points. Higher MISS scores were associated with significantly higher rates of spontaneous vaginal delivery, vaginal birth after cesarean, and breastfeeding, and significantly lower rates of cesarean, preterm birth, low birth weight infants, and neonatal death. MISS scores also correlated with density of midwives and access to care across birth settings. Significant differences in newborn outcomes accounted for by MISS scores persisted after controlling for proportion of African American births in each state.

**Conclusion:**

The MISS scoring system assesses the level of integration of midwives and evaluates regional access to high quality maternity care. In the United States, higher MISS Scores were associated with significantly higher rates of physiologic birth, less obstetric interventions, and fewer adverse neonatal outcomes.

## Introduction

The Lancet Series on Midwifery (2014) concluded that “national investment in midwives and in their work environment, education, regulation, and management … is crucial to the achievement of national and international goals and targets in reproductive, maternal, newborn, and child health” [1]. In countries where midwives are integrated into the health care system, the benefits of midwifery care are well-documented [2]. Global health experts recommend scaling up midwifery to improve maternal and newborn outcomes, reduce rates of unnecessary interventions, and realize cost savings [3,4]. However, access to midwifery care in the United States (US) is markedly lower than in most other “Organisation for Economic Co-operation and Development” (OECD) countries, with approximately 10% of US births attended by midwives compared to 50–75% in other high-resource countries [5]. In addition to low density of midwives per state, all midwives are not universally licensed to practice or integrated into regional health care systems. American midwives face multiple challenges to practice, including numerous regulatory barriers and inability to secure third party reimbursement [6]. As a result, women in many states cannot access midwives because of legal or payor restrictions [7,8].

Regulation has been identified by the International Confederation of Midwives as one of the pillars of a strong midwifery profession [9]. Regulation refers to a set of criteria and processes arising from the legislation that describes the scope of midwifery practice (activities which midwives are educated for, competent in, and authorized to perform, consistent with the ICM Definition of the Midwife) [9]. On a global scale, maternal and perinatal outcomes are better in jurisdictions where midwives are regulated and have the legislative authority to practice to their full scope across birth settings, including collaborating with or referring to other health professionals [2]. To date, it has been difficult to examine the impact of variations in midwifery regulation and integration across the United States on perinatal outcomes or on consumer access to maternity care. To address these gaps, a panel of maternity care and health policy experts who were delegates to the Home Birth Summit III [HBS] in 2014 (http://www.homebirthsummit.org/) designed **The Access and Integration Maternity Care Mapping (AIMM) Study.** The aim of this transdisciplinary, national research project was to examine the impact of state regulatory environments on access to midwives and association with perinatal outcomes across populations in the United States.

### Why does integration matter?

There are very few jurisdictions in the United States (US) where all types of midwives, irrespective of practice site, are fully integrated as regulated health professionals into interprofessional care provider networks. However, interprofessional teamwork is essential to the provision of high-quality maternity care [10]. For example, research indicates that, when professionals collaborate on decision-making and when coordination of care is seamless, fewer intrapartum neonatal and maternal deaths occur during critical obstetric events [11]. Poor communication, disagreement, and lack of clarity around provider roles are identified as primary determinants of these adverse outcomes [10–12]. Beliefs about risk, beneficence, non-maleficence and patient autonomy are often discipline-specific and divergent [13,14]. Rates of intervention, and labour management options that facilitate normal, physiologic birth are known to differ by type of provider [15], by birth setting [16,17], and by provider education. When differences around defining risk and responsibility exist among providers, interprofessional cooperation and access to options for care are reduced [18–20]. Moreover, when patients perceive interprofessional conflict, the culture of safety is diminished [21–23].

Conversely, collaboration among health professionals can improve safety and quality, particularly when care is transferred from low to high resource settings [10]. For example, when a woman plans to give birth in a community setting (home or birth center) she benefits when her midwife can facilitate access to specialized hospital personnel, equipment, or medications when necessary. The ability of midwives to function autonomously to their full scope of practice in community settings, in collaboration with other members of the health system, can enhance cost-effectiveness of maternity care [24,25]. Regardless of birth setting, midwife-led care has been linked to significantly improved perinatal outcomes, and maternal experience, in both healthy and at-risk populations [26–28]. In the US, current evidence suggests that scope of practice laws, as well as other aspects of state policy and regulation, may be reducing the maternity care workforce and access to services [26]. An integrated maternity care system facilitates the full exercise of scope of practice, autonomy, self-regulation, and collaboration across disciplines.

### The diverse context for American midwifery practice

Over 15 years ago, the American Public Health Association issued a position statement, calling for increased access and integration of midwifery services in the United States. [29](29) Yet, consistent U.S. standards for regulation, scope of practice, and access to reimbursement for midwives are still lacking, resulting in a fragmented system of care.

There are three professional designations for midwives in the United States: Certified Nurse-Midwife (CNM), Certified Midwife (CM) and Certified Professional Midwife (CPM). CNMs/CMs obtain their basic education in midwifery through university-based nursing programs and obtain a master’s degree. Both CMs and CPMs are direct-entry midwives without a prior nursing credential. CPMs have a median of three years of education before attending deliveries as a primary midwife; half gain certification via portfolio review, 40% graduate from an accredited school and others report blended education pathways [7]. CNMs can obtain licensure in all 50 states and DC, and their scope includes well-woman gynecology and primary care, as well as maternity care. They are prepared for practice in any birth setting, but they almost exclusively practice in hospitals [6,30]. CMs are currently licensed in 5 states, and are prepared for an identical scope of practice and settings for care as CNMs. CPMs can currently obtain licensure in 30 states. They provide antepartum, intrapartum, and postpartum/newborn care in community based settings, but typically cannot obtain hospital practice privileges and often have difficulty establishing reliable systems for referral and collaborative care. [7,31]

Wide variations in state regulatory conditions for midwifery practice, especially with respect to birth place, have created an environment of interprofessional hostility in some jurisdictions and interprofessional cooperation in others. Given the emerging evidence on the adverse impact of interprofessional disarticulation on maternal experience and outcomes [31,32], it is important to understand the connections between different regulatory environments and differences in health outcomes, especially when significant disparities exist across populations. Differences in adverse perinatal outcomes between Caucasian women and women of colors are well-documented [33–36], and persist even when controlling for socio-economic status and access to quality prenatal care [33,37]. There is a dearth of information about whether health disparities can be attributed to differences in health insurance coverage, or access to providers, or quality of care [36,38,39].

In 2015, 89.8% of US births were attended by physicians, 8.5% by CNMs/CMs, 0.8% by other midwives (including CPMs), and 0.8% by other providers [40]. In 2014, methods of payment varied by place of birth: 44.2% of hospital births were paid for by Medicaid, 48.0% by private insurance, 3.4% were self-pay, and 4.4% via other sources. In contrast, 16.4% of community births (birth center or home) were paid for by Medicaid, 29.4% by private insurance, 50.0% through self-pay and 4.2% via other sources. Most community births are attended by midwives and half are not covered by insurance [41]. The regulatory environment for payors has been shown to significantly impact the extent of midwifery practice in a state and autonomy of midwives [42].

Such systems-level deficits may have significant, negative impacts on the health and well-being of maternal-newborn populations. Rates of obstetric interventions are on the rise in the United States and adverse maternal and newborn outcomes are high, compared to other OECD countries [43]. Black Americans experience substantially higher rates of maternal and neonatal mortality, preterm birth, and low birth weight [33,34,44]. However, one study found that in states where CNMs have greater professional autonomy (i.e. physician supervision not required), there were lower rates of surgical birth, preterm birth and low birth weight, even when adjusted for maternal age, parity, race, education, marital status, cigarette use and prenatal care utilization [26].

In the **Access and Integration Maternity Care Mapping (AIMM) Study,** we went beyond CNM autonomy to create an evidence-based scoring system to rank the level of integration of all types of midwives into health systems. We then examined the relationships between state Midwifery Integration Scores, density of midwives, access to midwives across practice settings, rates of obstetric interventions, and maternal and newborn outcomes.

## Methods

We convened a multi-disciplinary Task Force with expertise in maternity services research, public health, midwifery, obstetrics, epidemiology, consumer advocacy, and/or roles in midwifery regulation, legislation, and law. They identified the key variables needed to populate a database of published regulatory data across all 50 states and the District of Columbia, detailing rules regarding scope of practice, and requirements for licensure of CNMs, CPMs, and CMs and practice across birth settings. We then employed a formal, process **(see Table 1)**, modeled on the Delphi method [45,46], best practices for transdisciplinary research, and legal epidemiology [47], to identify and validate the most important items for inclusion in a composite measure of midwifery integration.

**Table 1:** Development of an evidence-based Midwifery Integration Scoring System (MISS).

**Delphi Round 1 –Concept generation**
• HBS Regulation and Licensure Task Force (Team 1) reviews source documents and identifies 7 domains of midwifery integration • Database populated with state regulations on scope of practice and restrictions • Team 1 agrees by consensus on 110 key items describing midwifery regulation
**Delphi Round 2 –Expert content validation**
• HBS Research and Data Task Force (Team 2) defines optimal regulatory conditions that support patient access and collaborative practice–informed by a review of the evidence, and consultation with Team 1 • Database and rubrics translated into format to allow for a ranked composite scoring and comparison across states • State regulatory content experts (N = 92, 1-2/state) review items and scoring rubrics for accuracy and relevance to local implementation of the law • Team 2 harmonizes data and adapts scoring rubrics to reflect state realities • Final scoring system reviewed and confirmed by consensus among Teams 1 and 2, and national midwifery regulators and clinical leaders
**Delphi Round 3 –Development and application of composite measure**
• Team 2 selects 50 key indicators of midwifery integration indicating level of autonomy, ability to practice to full scope, and collaboration across birth settings. • Teams 1 and 2 convene to rank order answer options in each of the 50 items (higher scores indicated more favourable access and practice conditions) • Team 1 develops a weighted scoring system based on patient safety and quality. Item level scores are weighted and summed for a total optimal score of 100. • MISS tool generates State Integration Scores (range = 17 to 61 across the US). • Density of midwives (per 1000 state births) and access to midwives across settings (home, birth center, hospital) correlated to MISS scores and outcomes. • Correlation and regression analyses link state MISS scores to selected perinatal outcomes that are reliably reported by CDC Vital Statistics
**Delphi Round 4 –Development of the AIMM report card**
• Teams 1 and 2 meet to reach consensus on interpretation and key messages • Creation of Interactive AIMM Maps: ○ MISS scores categorized into four quartiles (very low, low, moderate, high)^1^ ○ Perinatal outcomes linked to MISS scores and displayed by highest and lowest quartiles ○ 4 base maps to display: level of integration, density, proportion of midwife-attended births in 3 settings, and proportion of black births by state

**1:** We categorized MISS scores and outcomes into four equal categories: Values between the 1-24^th^ percentile, the 25^th^-49^th^ percentile, the 50^th^ to 74^th^ percentile and the 75^th^ to 100^th^ percentile.

### Round 1 –Concept generation

The Task Force self-organized into two teams, one with regulatory, law, and consumer access expertise, and another with expertise in public health, legal anthropology, and perinatal epidemiology research methods, including instrument development. Both teams included clinicians, and consumers. Over three rounds of drafts, edits, and consensus-based discussions, Team 1 identified seven relevant domains that were important to identify in state regulations on midwifery. Four domains describe midwifery practice: scope of practice, provider autonomy, governance, access to referral and medications; and three domains describe patient safety, quality, and access to maternity providers across birth setting. The team identified 110 indicators that differentiate the regulatory environment by domain for each type of midwife (CM, CPM, or CNM), and assigned numeric values to describe the diverse conditions, permissions, or restrictions delineated in the state laws **(see Table 2).**

**Table 2:** Sample midwifery integration indicators and weighted scores.

**Are CPM/CNM/CMs regulated?** • 0 = Prohibited • 1 = Allowed by previous judicial opinion or not mentioned/not prosecuted to date • 2 = Unregulated but allowed by statutory permission • 4 = Licensed
**Are there statutory limitations/restrictions to site of practice for licensed CPM/CNM/CMs?** • 0 = Yes • 1 = Lack of access to hospital privileging or physician referral/signer • 2 = No
**Consultation/referral required by law for certain conditions?** • 0 = Unregulated state • 1 = Required (R) but difficult to access when needed • 2 = Not required (NR) but difficult to access when initiated by midwife • 3 = R or NR but easily accessed when initiated by CPM/CNM/CM
**Evidence-informed, validated quality assurance (QA)/quality improvement (QI) state system for all sites (home, hospital, birth centers)** • 0 = Hospital only • 1 = Hospital and birth center only • 4 = Home/hospital/birth center
Is Medicaid reimbursement available for CPM/CNM/CMs? • 0 = No • 2 = Yes, but challenges with reimbursement including birth site • 3 = Yes
**Do CPM/CNM/CMs have prescription-writing authority?** • 0 = Prohibited or not authorized • 1 = Allowed only by physician • 2 = Limited list of medications allowed • 3 = Comprehensive list of medications allowed • 4 = Prescription-writing authority

We then widened the consultant pool to include experts from national regulatory, legal, payor, professional and perinatal surveillance bodies. These policy leaders noted that the statutory language does not always accurately represent the realities of how rules and laws are interpreted and implemented. Language used in rule-making may be interpreted in more or less restrictive ways, and some rules are not actionable given infrastructure constraints and systems-level limitations. For example, in one state, CPMs have statutory authority to access emergency medications for the management of complications, such as maternal hemorrhage; however, pharmacists in that state are restricted from furnishing these medications to practitioners who are not affiliated with hospitals. Because CPMs cannot gain access to hospital privileges, they must find alternate ways to exercise their authority to carry these lifesaving medications.

### Round 2 –Expert content validation

Hence, to verify the realities of implementation of the law within each state, Team 2 identified and recruited state and national regulatory experts (n = 92) to complete an online survey. Participants included 75 state-specific regulatory board representatives; the presidents, regional and chapter chairs for state midwifery associations, state legislative and policy chairs for the American College of Nurse Midwives (ACNM) and National Association of Certified Professional Midwives (NACPM). They evaluated the connections and discordances between theory and practice for each of the identified indicators within the state regulatory environment. In poorly integrated states our national experts (ACNM, NACPM legislative directors) referred us to local midwifery or consumer experts who could reliably speak to ‘on the ground’ conditions. When two state experts disagreed on an indicator or experts did not know the answer, we further consulted with 17 state or national regulators, to resolve discrepancies.

We harmonized expert responses with our regulatory database through a systematic line-by-line comparison. We validated and/or deferred to the statutory language when there were no discrepancies between statutes and local interpretation or implementation. When state experts provided evidence of local interpretation that differed from the apparent intent of laws or rules, we added or adapted response options to reflect the realities of midwifery practice, consumer access, and/or the interprofessional environment.

### Round 3—Development and application of composite measure

A final Delphi process **(see Table 1),** involving both multidisciplinary teams, led to selection of 50/110 indicators of midwifery integration, and the development of a weighted Midwifery Integration Scoring System (MISS) (50 items, maximum summary score 100) that quantifies the potential impact on patient access to high-quality maternity care across birth settings. Both teams reviewed the 110 items and only retained those that were deemed, by consensus, important or very important to the assessment of midwifery integration. In some cases, 2–3 items were combined into one stem query, and response options expanded. Some items were excluded because team members felt that the items were not directly pertinent to midwifery integration. For example, one item (Does informed consent language in statute and/or regulations allow for informed refusal by client?) was excluded because the item relates more to human rights issue rather than quantifying the level of midwifery integration. To create the weighting system, using a scale of 0 (not important), 1 (somewhat important), 2 (important), 3 (very important), 4 (essential), the teams assessed each item for its potential impact on patient access to high-quality maternity care. They assigned higher item-level scores to indicators of greater integration, more interprofessional collaboration, and/or wider consumer access across birth settings. The final list of items describe the range of possible options for scope of practice, regulatory body, prescriptive authority, requirements for physician supervision, access to Medicaid, etc. that vary in both statutory language and implementation across states. See S1 Table for a full list of the indicators and scoring system.

### Ranking states by MISS scores and outcomes

We used the MISS composite summary scores to rank states by degree of integration. Then, using the 2014 CDC-Vital Statistics Database, we calculated Spearman’s rho correlation coefficients between the continuous MISS integration scores and selected maternal-newborn outcomes in each state. We used Spearman’s rho because the MISS scores were normally distributed as indicated by the Shapiro-Wilk Test (0.960, p = 0.08), but the outcomes data were not. We selected indicators that represent cost-effectiveness and quality in perinatal care (e.g. rates of spontaneous vaginal birth, exclusive breastfeeding, cesarean, induction, VBAC, preterm birth, low birth weight, neonatal mortality) [43,48], and were available and reliable in the CDCs Vital Statistics database [47]. Finally, based on data from the Area Health Resource File, and Centers for Medicare and Medicaid Services, we calculated correlations between MISS scores; state density of midwives (per 1000 births); and consumer access to midwives across birth settings, defined as the proportion of all births at 1) hospital, 2) home and 3) birth centers for two categories of midwives a) CNMs/CMs and b) CPMs and other direct entry midwives as reported on the birth certificates for each state.

In addition, we calculated the correlations between 1) CM and 2) CPM licensure and perinatal outcomes, to examine the differential effects of licensure versus integration scores by state for all outcomes. We also identified states with the highest increases in community births (at home and birth centers) over the past 8 years and examined correlations with MISS scores.

Finally, appreciating the complex nature of health disparities, to understand the relative importance of midwifery integration on perinatal outcomes, we conducted hierarchical linear regression modelling, to control for the proportion of Non-Hispanic Black births in each state, when examining the relationship of MISS scores with rates of five outcomes: caesarean, preterm birth, neonatal death, low birth weight, and breastfeeding at birth.

## Results

**Fig 1:**
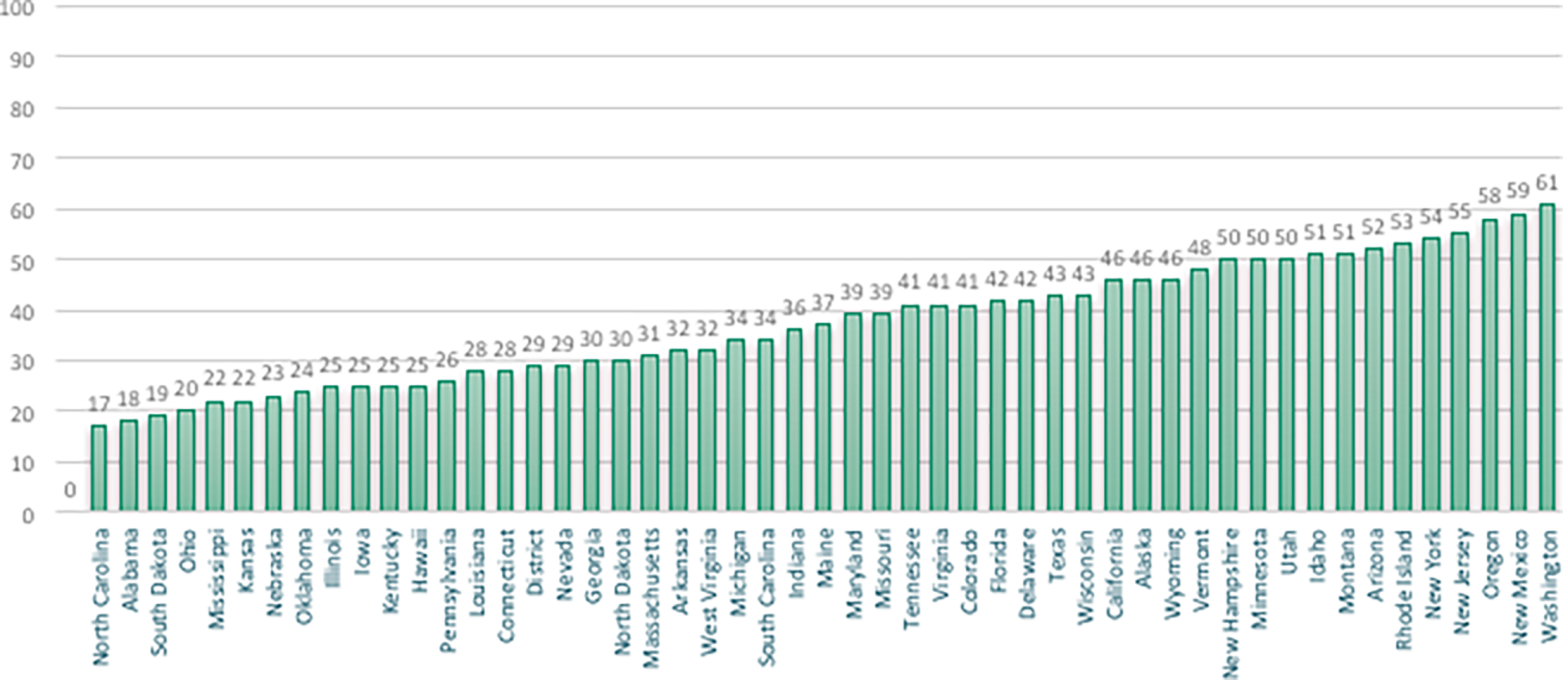
Rank-ordered integration scores for 50 states and Washington, DC (2014–2015).

**Fig 2:**
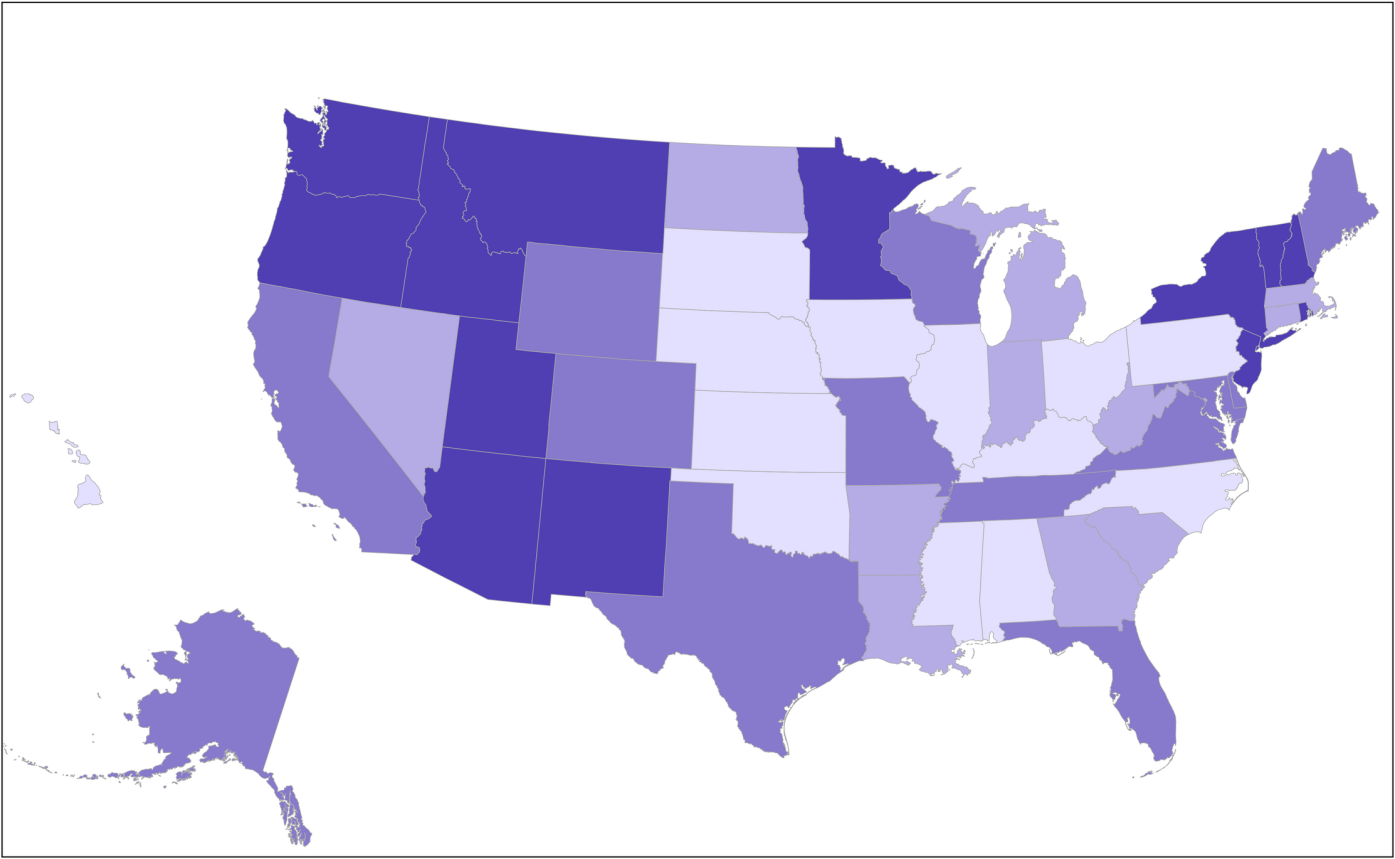
Map of midwifery integration across the United States. Levels of integration displayed by quartiles of MISS scores. Deeper shades of purple represent higher integration and lighter shades represent lower integration of midwives.

State MISS scores ranged from 17 in North Carolina to 61 in Washington State, with notable regional variation **(see Figs 1 and 2)**. Higher MISS integration scores were correlated to a higher density of midwives per state and higher proportion of midwife-attended births across settings **(see Table 3)**. Higher MISS scores, and improved access to midwives in all settings, were associated with significantly higher rates of spontaneous vaginal delivery, vaginal birth after cesarean (VBAC), and breastfeeding at birth and at six months; and significantly lower rates of cesarean section (CS), preterm (PTB), and low birth weight (LBW) infants **(see Table 4)**. Higher MISS scores were correlated strongly with lower rates of neonatal mortality **(see S1 Fig)** and race-specific neonatal mortality **(see S2 Table).**

**Table 3:** Significant correlations between MISS scores, and density and access to midwives by setting, United States, 2014.

State-level	Correlation coefficient
Density of CNMs/CMs (per 1000 births)	0.495**
Density of CPMs (per 1000 births)	0.459**
Proportion of midwife-attended births all locations	0.431**
Proportion of midwife-led births in community settings	0.509**

**Correlation is significant at the 0.01 level (2-tailed).

Notes: Midwifery density was determined separately for CNMs/CMs and for CPMs by dividing the number of midwives in each category in each state by the total number of births in each state and multiplying by 1000. Consumer access to midwives across birth settings was defined as the proportion of all births documented at 1) hospital, 2) home and 3) birth centers for a) CNMs/CMs and b) CPMs and other direct entry midwives as reported on the birth certificates for each state.

**Table 4:** Significant correlations between midwifery care, MISS scores, and birth outcomes, United States, 2014.

%	% of births attended by all types of midwives, hospital only	% of births attended by all types of midwives in community birth settings	Midwifery Integration State Scores
Spontaneous Vaginal Birth^1^	0.556**	0.435**	0.402**
Vaginal birth^1^ after Cesarean^2^	0.483**	0.528**	0.330*
Induction^3^	-0.350*	-0.084	-0.275
Preterm birth^4^	-0.556**	-0.455**	-0.480**
Low birth weight^5^	-0.299*	-0.388**	-0.353*
Cesarean section^2^	-0.375**	-0.627**	-0.278*
Neonatal mortality rate^6^	-0.247	-0.364**	-0.545**
Breastfeeding at birth	0.474**	0.593**	0.584**
Breastfeeding^7^ at 6 months	0.524**	0.533**	0.378**

**Correlation is significant at the 0.01 level (2-tailed).

*Correlation is significant at the 0.05 level (2-tailed).

^1^ a vaginal birth without prior induction.

^2^ all types of Cesarean sections.

^3^ all types of inductions.

^4^ births before 37 weeks gestation.

^5^ babies weighing less than 2500 grams at birth.

^6^ babies that died within 27 days of birth per 1000 births in the year 2013.

^7^ exclusive breastfeeding.

Source: Authors, analysis of MISS scores, and data from CDCs Vital Statistics database (2014), 2013/ 2014 National Immunization Surveys and Area Health Resource File. Data for breastfeeding at 6 months is for the year 2012 and was obtained from the 2013 and 2014 National Immunization Surveys: https://www.cdc.gov/breastfeeding/data/nis_data/rates-any-exclusive-bf-state-2012.htm.

Between 2004 and 2014, community birth rates increased significantly (p< 0.05) in all states, except Vermont, Rhode Island, Oklahoma, Maine and DC. The average increase over the time period was 72%. [41] The states with the largest increases were Montana, Oregon, Washington, Utah and Wyoming. States with higher MISS scores had significantly higher rates of community births in 2014 (r_s_ = 0.445, p = 0.01) and significantly larger increases in community birth rates from 2004–2014 (r_s_ = 0.328, p = 0.02).

Our regulatory data described conditions for 2014–2015, when CPMs had regulatory authority to practice in 27 states and CMs in 5 states. CPM licensure significantly correlated to access to midwifery care in community settings (r_s_ = 0.440, p = 0.001). Licensure alone was not synonymous with integration, and did not confer the same benefits on outcomes or interventions (**see S4 Table**).

MISS scores were significantly lower in states with a higher proportion of non-Hispanic Black births (r_s_ = - 0.370; p = 0.007). Access to midwives across settings and density of midwives were also significantly lower in states with a higher proportion of black births (r_s_ = - 0.375, p = 0.007 and r_s_ = - 0.298, p = 0.04). To determine the amount of variance that is accounted for by integration of midwives, when taking into account disparities in neonatal mortality by race, we undertook further analysis. Differences in the percent of Non-Hispanic black birth across states accounted for 38.5% of the differences in neonatal mortality scores, and MISS scores explained another 11.6% of variance (**see Table 5**). This change was significant (p = 0.002) meaning that the level of integration can explain differences in neonatal mortality rates, above and beyond the percent of black births. These two factors, state-level percent of black births and level of midwifery integration, can predict half of the variance (50.1%) in neonatal mortality rates. MISS scores also explained significant additional variance in rates of preterm birth and breastfeeding at birth scores (**see Table 5**). Integration scores did not add significant explanatory power to disparities in cesarean and low birth weight rates.

**Table 5:** Results from linear regression analysis, showing variations in outcomes that can be explained by % black births and MISS scores.

	Outcome	Variance explained by % black birth (R2)	Additional variance explained by MISS integration scores (R2)	Total variance explained
**Model 1**	Neonatal death	0.385	0. 116*	0.501
**Model 2**	Cesarean section	0.427	0.006	0.433
**Model 3**	Preterm birth	0.371	0.081*	0.452
**Model 4**	Low Birth Weight	0.552	0.018	0.570
**Model 5**	Exclusive breastfeeding at birth	0.425	0.107*	0.532

*R square change significant (< 0.05).

Regression specifications: Hierarchical linear regression. The proportion of black births was entered in block 1 of the model and integration scores in the second block; outcomes were: Neonatal death, preterm birth, low birth weight, CS and breastfeeding at birth. For each model we found that the relationship between standardized predicted values and standardized residuals was linear and that the observed standardized residuals were normally distributed. A p value < 0.05 was deemed as significant.

## Discussion

Our analyses showed that a state regulatory environment that supported greater integration of midwives into the health system was associated with a greater number of midwives and midwife-attended births in a state. This greater integration was significantly associated with higher rates of spontaneous vaginal birth, VBAC and breastfeeding at birth and at six months, as well as lower rates of obstetric interventions, preterm birth, low birth weight infants, and neonatal death. These findings are especially significant in the light of increased costs to any health care system associated with high cesarean and preterm birth rates, and low breastfeeding rates. This is a pathognomonic example of the current global public health focus on, “too much too soon, too little too late” [48].

While the associations illustrated do not imply causation, the MISS scores nonetheless can be a tool for generating testable hypotheses on the effects of midwifery integration on key outcomes. The observed relationships may help us identify states where childbearing women are at increased risk for poor outcomes and experience reduced access to high quality maternity care due to poor integration of midwives across birth sites (e.g. North Carolina, Alabama).

Model states (i.e. states with the highest MISS scores) can inform mechanisms to enhance integration of midwives in other states. However, it is important to note that no state enjoys regulatory conditions that are optimal to support quality and safety for families during pregnancy, birth and the postpartum period. The most integrated states now achieve scores that represent less than two thirds (61/100) of condition requirements for a fully integrated system for care.

### Can integration of midwives reduce health disparities?

African American mothers, in particular, are affected by adverse maternal and newborn outcomes; they experience a two to four times higher risk than White women for both maternal and infant mortality [44,49]. Some policy makers and public health experts attribute this to concomitant disparities among African Americans in wages, housing, and safe environments. Other researchers have proposed that outcomes such as LBW are due, in part or wholly, to experiences of discrimination across the life span of African-American mothers [50,51]. Giscombe and Lobel [52] hypothesized that racism functions as a severe stress trigger, and have explored biologic explanations for how stress influences adverse neonatal outcomes.

Nonetheless, Rossen et al. [53] describe considerable variation in county-level and race-specific infant mortality rates between black and white mothers. They suggest that this variation might be partly a result of contributing factors that are common to both white and black infants, including differential access to specialized care, perinatal regionalization, and overall patterns in health care delivery. Since data suggest that institutional racism is a contributing factor, place of birth, or model of maternity care, may also modulate these outcomes [41]. A recent population-level analysis in Canada described associations between midwifery care of at-risk populations and significantly reduced incidence of pre-term birth, low birth weight, and other adverse outcomes [54]. In our study, lower MISS scores were associated with significantly higher rates of neonatal mortality among Hispanic, black and white babies when examining race-specific outcomes. Density of midwives and access to midwives across birth settings were also significantly lower in states where more black babies are born. The additional variance explained when MISS scores were added to the equations suggests that, with greater integration of midwives in these states, the associated reduced rates of neonatal mortality, preterm birth, and increased breastfeeding success could confer important long term health benefits [55,56] for African American mothers.

### Does midwifery integration affect outcomes across birth settings?

Some investigators have suggested that poor neonatal outcomes may rise with increased access to midwives who attend home and birth center births [57,58]. In our state-by-state comparison, however, the best outcomes for mothers and babies occur in states where all types of midwives are regulated and integrated into the health care system regardless of birth setting.

Nonetheless, significant interprofessional conflict persists around recommendations for safe birth care. For example, despite the emergence of high quality prospective observational studies supporting the safety and cost-effectiveness of planned home birth [16,24], leading maternity providers in North America have been in conflict about birth at home and birth centers, debating issues related to safety, access, the value of obstetric intervention, and patient autonomy [57–59].These debates are reflected in widely varying state regulatory environments that may, in turn, influence conditions for maternity practice and public access to choice of birth place. Differences in community birth rates across regions may simply represent the influence of pre-existing provider philosophies and attitudes [60], which in turn affect informed consent discussions with patients as well as comfort with collaboration across disciplines [20].

Rates of planned home and birth center birth in the US and Canada remained at less than 1% for several decades, but current data suggest that American women are increasing their interest in this option [41]. Midwives are the only maternity care providers who currently offer choice of birth setting. However, because not all types of American midwives can legally practice in all birth settings, choice of birth place is functionally quite limited for a majority of US women. In some regions, women who plan to deliver at home or in a birth center, will (along with their midwives) encounter hostility, judgment, and, reprimand when they transfer across birth settings [31,61]. Person-centred maternity care should define quality and safety within a multi-faceted context that includes patient choice, access, experience, and cost-effectiveness.

### Policy implications: Improving access to high quality maternity care

Our ranking system highlights discrepancies in integration and related outcomes and could inspire political will, and guide legislative reform. The Midwifery Integration Scoring System can help to identify states where childbearing women and newborns might benefit from improved integration of midwives. In communities where access to ***any*** maternity provider is scarce [62], our findings suggest that expanding access to midwifery care may be an important part of the solution to both public health and human health resource challenges. All three types of midwives share a model of maternity care that has been associated with optimal outcomes and cost-effectiveness [1,63]by prioritizing person-centered care; promoting of normal, physiologic birth; judicious evidence-based use of obstetric interventions and procedures; and collaborating with and/or referring to obstetric specialists when indicated [7,64]. Our results align with this evidence suggesting that increased reliance on midwives could reduce the costly overuse of obstetric interventions, reduce rates of preterm birth and neonatal loss, and improve breastfeeding and vaginal birth rates, thereby helping to address serious maternal-newborn health deficits in the United States.

The US precedent of health systems restricting access to qualified attendants across birth settings, and placing high value on institutional birth, has been very influential in low and middle resource countries. [65,66]Unfortunately, the system of incentivizing institutional birth and physician management of healthy pregnancies has exacerbated the gaps between demand and available health human resources both in the US and in low resource settings. [66,67]Skilled midwives can assist a woman to assess her birth site options according to her health status and facilitate access to appropriate resources. Ideally, they would practice in a legal environment that allows them to practice to full scope, and collaborate seamlessly with other health professionals, across birth settings.

To enable midwives to work autonomously within their full scope of practice, the International Confederation of Midwives has identified standards for regulatory mechanisms that protect the public by ensuring that midwives provide high quality midwifery care to every woman and baby [68]. Based on these ICM Standards, the US Midwifery Education, Regulation and Association (USMERA) workgroup has described Principles for Model Midwifery Legislation [69]that include many of the same components that comprise the MISS scores. If applied to state regulatory reform, they could contribute to state scores that are closer to the ideal (i.e. 100).

A recent Lancet analysis of maternal health policy revealed that countries with a sustained 20-year decrease in maternal mortality had increased country-wide access to health care through targeted investment in midwifery services. [4] In countries like India, Mozambique, Uganda, and Nepal skilled birth attendants are scarce in all settings and the consequences are disastrous–“too little too late” [48]. In high resource countries that are experiencing the phenomena of “too much, too soon”, expanding availability of midwives across health systems also has important implications for quality, safety, and cost-effectiveness [43,48].

## Limitations and opportunities

While this analysis represents a significant step forward, it has some limitations. We are using aggregated state measures and hence potentially subject to the ecological fallacy of making inferences concerning individual behavior, based on group data [70]. However, our goal is to measure systems of care at the state level rather than the relationship between individual providers and specific neonatal outcomes. We cannot conclude that a more integrated system of midwifery directly causes improved outcomes. It may simply reflect a state culture of better interprofessional cooperation that affects patterns of practice. Variations in access to *any* maternity care at the local level may have more impact on outcomes, and data derived from Area Resource maps on provider availability may be more informative. Functional levels of integration may vary by the interpretation of statutes by providers or referral institutions at the local level.

Our analysis captured relationships as relevant to the regulatory environment in the US in 2014–2016. As regulatory and practice conditions change, MISS scores will also change, so ongoing revisions of the source database will be necessary. Our team plans to partner with NACPM, ACNM, and regulatory boards to tri-annually update the data-informed AIMM maps.

Our findings could inform site selection for a national prospective cohort study, such that studies of midwifery outcomes can be restricted to states with high MISS scores or can control for level of integration. Cohort studies that take into account the level of midwifery integration could inform state regulatory language that supports increasing access to high quality care across settings and jurisdictions.

Finally, the MISS scoring system is based on evidence-based metrics that are relevant to midwifery regulation and practice globally. This composite scoring system could be adapted to country-level realities where items describe the domains according to the available maternity providers and regional conditions for practice, restrictions, and state of collaboration. It is likely that other high resource countries would achieve scores that represent a more fully integrated system, consistent with their reported improved outcomes.

The 2014 Lancet Series on Midwifery, in collaboration with the WHO, identified the top 11 research priorities needed to improve quality maternal and newborn care. [71] Global experts recognized that it is critical to ask “different questions” if we are to understand which outcomes are most important to track and which factors most contribute to those essential outcomes. To fully understand the relationships between health systems, model of care, access to care, and childbearing outcomes, more investigations on the impacts of the regulatory environment at the local, regional, and country level is needed.

### The AIMM Report Card

**Fig 3:**
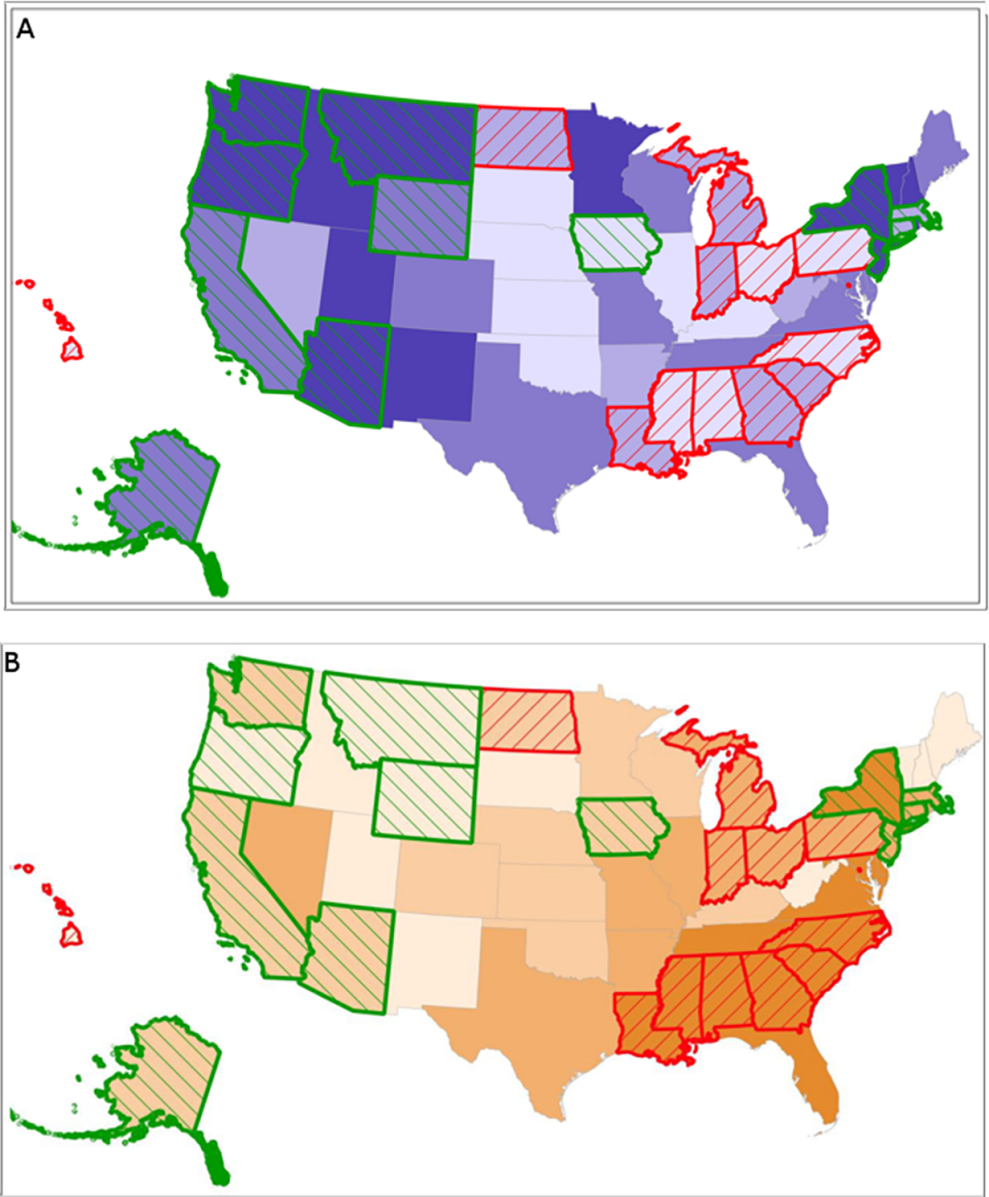
Base maps showing integration and percent of black births with neonatal mortality quartiles highlighted. 3A: Levels of integration displayed by quartiles of MISS scores. Deeper shades of purple represent higher integration and lighter shades represent lower integration of midwives. Green outlines show where rates of neonatal mortality are lowest and red outlines show where rates are highest. 3B: Percent of black births per state by quartiles. Deeper shades of orange represent a higher proportion of black births and lighter shades represent a lower proportion of black births. Green outlines show where rates of neonatal mortality are lowest and red outlines show where rates are highest.

To make our findings more accessible to policy makers and consumers, Team 2 worked closely with a GIS specialist to create the AIMM “Report Card”, a visual representation of the data via a series of color-coded, interactive maps. The maps illustrate the range of midwifery integration across the United States by quartiles, as well as density and access to midwives in different settings. The AIMM Report Card displays how integration, access, and density of midwives link to outcomes by distinguishing states that are in the highest and lowest quartiles for indicators of optimal health according to global health agencies (e.g. WHO-recommended rates for cesarean). For example (**see Fig 3A & 3B**), on each map, upon selection of outcomes, green outlines appear for states that report the highest rates of spontaneous vaginal birth, vaginal birth after cesarean (VBAC), and breastfeeding. Red outlines appear for states in the highest quartile for rates of cesarean, induction, neonatal mortality, prematurity, and low birth weight infants.

The maps also show race-specific outcomes by MISS scores, and display outcomes by the proportion of Black women giving birth in each state. **Fig 3A** displays that, in most states where black women give birth, they do not have access to midwives who are well integrated into the system. These states also report the highest rates of neonatal mortality. New York State is a notable exception, reporting a high density of black births, among the lowest rates of neonatal mortality in the country, and a MISS score in the highest quartile.

Through the AIMM Report Card, the viewer can visualize how perinatal outcomes, interventions, and access to choice of birth place differ in states where midwives are well integrated, compared to states where disarticulations exist. Similarly, pop-up bar and pie graphs display state-level data for the proportion of women giving birth by settings, proportion of births attended by midwives that are covered by Medicaid, and state level rates of perinatal outcomes.

The data maps are available at http://birthplacelab.org/maps. State-specific report cards can be viewed at http://www.birthplacelab.org/how-does-your-state-rank.

These tools may be valuable to advocates, policy makers and other key stakeholders who seek to identify regions with reduced access to collaborative practice and options for maternity care.

## Conclusions

The Midwifery Integration Scoring System (MISS) is a powerful new tool to track the impact of the regulatory environment on patient access to health care, as well as choice of provider and birth place. The Access and Integration Maternity care (AIMM) Maps illustrate effective health human resource allocation in maternity care, based on population-level health outcomes data. Higher MISS Scores were associated with significantly more access to midwives, significantly higher rates of physiologic birth outcomes, lower rates of obstetric interventions, and fewer adverse neonatal outcomes. Race is associated with significant differences among states in neonatal outcomes; and the level of integration of midwives accounts for additional differences that persist after controlling for African American births. Our findings can inform health policy to improve regional access to high quality maternity care across populations and birth settings.

## Supporting information

S1 Table Midwifery Integration Scoring System (MISS) indicators. (DOCX)

S2 Table Association between MISS scores and state-level, race-specific neonatal death rates. (DOCX)

S3 Table Correlations between licensure, outcomes, and interventions. (DOCX)

S1 Fig Scatter plot showing relationship between integration scores and neonatal death (2013). (DOCX)
