# Supplementary material for: Mapping integration of midwives across the United States: Impact on access, equity, and outcomes
Source: PLoS One. 2018 Feb 21;13(2):e0192523. doi: 10.1371/journal.pone.0192523 (PMC5821332; doi:10.1371/journal.pone.0192523)
Supplement: S1 Table — (DOCX) [file pone.0192523.s001.docx]

**S1 Table: Midwifery Integration Scoring System (MISS) indicators**

| **OPTIONS FOR BIRTH SITE** | **Optimal**  **Score** |
| --- | --- |
| Do CPMs offer planned home birth services? 0=No or under legal duress; 1=Yes | **1** |
| Do CNMs offer planned home birth services? 0=No; 1=Yes | **1** |
| Do CMs offer planned home birth services? 0=No; 1=Yes | **1** |
| Do MDs offer planned home birth services? 0=No; 1=Yes | **1** |
| Do other care providers (e.g. Licensed midwife, Naturopathic Doctor, Doctor of Osteopathy, etc.) offer planned home birth services? 0=No; 1=Yes | **1** |
| Do CPMs offer birth center services? 0=No; 1=Yes | **1** |
| Do CNMs offer birth center services? 0=No; 1=Yes | **1** |
| Do CMs offer birth center services? 0=No; 1=Yes | **1** |
| Do MDs offer birth center services? 0=No; 1=Yes | **1** |
| Do other care providers (e.g. Licensed midwife, Naturopathic Doctor, Doctor of Osteopathy, etc.) offer birth center services? 0=No; 1=Yes | **1** |
| Are there statutory limitations or restrictions to site of practice for CNMs? 0=Yes; 1=Lack of Access to Hospital Privileging or Physician Consultation/Referral/Signer ; 2=No | **2** |
| Are there statutory limitations or restrictions to site of practice for licensed CPMs? 0=Yes; 1=Lack of Access to Hospital Privileging or Physician Consultation/Referral/Signer; 2=No | **2** |
| Are there statutory limitations or restrictions to site of practice for licensed CMs? 0=Yes; 1=Lack of Access to Hospital Privileging or Physician /Consultation/Referral/Signer; 2=No | **2** |
| Are there statutory limitations or restrictions to site of practice for MD? 0=Yes; 1=limits access to hospital privileges if attends home births, 2=No | **2** |
| Does state have evidence-informed, validated QA/QI state system for all sites (home, hospital, birth centers) ? Hospital only =0, hospital and birth center only = 1, home/hospital/ birth center = 4 | **4** |
| Are there statewide systems for smooth transfer across birth sites? 0=No, 3=Yes | **3** |
| **REPORTING AND DATA COLLECTION** |  |
| Does the birth certificate in the state record planned place of birth as well as actual place of birth? 0=No; 1=Yes | **1** |
| **VBAC** |  |
| Is VBAC allowed for licensed midwives? 0=Prohibited or unregulated state; 1=allowed only by restrictive conditions (eg physician approval); 2=allowed by meeting certain conditions and with informed consent; 3=unrestricted | **3** |
| **CERTIFIED PROFESSIONAL MIDWIVES (CPM)** |  |
| **Regulation & Medicaid** |  |
| Is direct-entry midwifery regulated? 0=Prohibited; 1=Allowed by previous judicial opinion/ or not mentioned/not prosecuted to date; 2=Unregulated but allowed by statutory permission; 4=Licensed | **4** |
| Is CPM credential sufficient for licensure (additional steps for licensure may be required: TB test, state application requirements, state examination, etc.)? 0 = No; 1 = Yes | **1** |
| Is Medicaid reimbursement available? 0 = No; 2 = Yes, but challenges being reimbursed; 3=Yes | **3** |
| **Autonomous Practice & Risk Assessment** |  |
| Is physician supervision or outside assessment required? 0=Yes; 1=No | **1** |
| Is a consultation agreement/collaborative practice agreement with physician required? 0=Yes, formal written agreement/or formal consultation req'd/or unregulated; 1=Yes, but informal & unwritten; 3=No agreement req'd | **3** |
| Is consultation/referral required (instead of recommended) by law for certain conditions? 0 = Unregulated state; 1=Required but difficult to access when needed; 2=Not required but difficult to access when initiated by CPM; 3=Required or Not required but easily accessed when initiated by CPM | **3** |
| **Scope of Practice** |  |
| Is scope of practice limited by law to childbearing year? 0 = Yes; 1 = No | **1** |
| Does scope of practice include well-woman care? 0 = No; 1 = Yes | **1** |
| **Medications - Authority of Midwife to Obtain and Administer Medications** |  |
| What level of prescription-writing authority do CPMs have? =4, Comprehensive list of medications given (specialized care medications - may require training, e.g. GBS prophylactic antibiotics, Pitocin) = 3; 2= Limited list of medications allowed (routine and emergency medications, e.g. newborn antibiotic eye ointment, anti-hemorrhagic drugs), 1=Allowed only by physician prescription, 0= Prohibited or not authorized | **4** |
| Do CPMs experience any challenges accessing any of the listed medications they are authorized to obtain and administer? 1= No; 0=Yes | **1** |
| **Midwifery Board/Council/Advisory Committee Composition & Regulatory Agency** |  |
| Is midwifery representation on Board/Council/Advisory group required? 0 = No; 1 = CPM not specified; 2 = Yes | **2** |
| **CERTIFIED NURSE MIDWIVES (CNM)** |  |
| **Regulation & Medicaid** |  |
| Are CNMs regulated? 1=No; 4=Yes | **4** |
| Is CNM credential sufficient for licensure (additional steps for licensure may be required: TB test, state application requirements, state examination, etc.)? 0 = No; 1 = Yes | **1** |
| Is Medicaid reimbursement available? 0 = No; 2 = Yes, but challenges with reimbursement including birth site; 3= Yes | **3** |
| **Autonomous Practice & Risk Assessment** |  |
| Is physician supervision for practice required? 0=Yes; 1=No | **1** |
| Is a consultation agreement/collaborative practice agreement with physician required? 0=Yes,formal written agreement/or formal consultation req'd/or unregulated; 1=Yes.but informal & unwritten; 3=No agreement req'd | **3** |
| Is consultation/referral required (instead of recommended) by law for certain conditions? 0 = Unregulated state; 1=Required but difficult to access when needed; 2=Not required but difficult to access when initiated by CNM for home and birth center; 3= Not required and easily accessed when initiated by CNM | **3** |
| **Scope of Practice** |  |
| Is scope of practice limited by law to childbearing year? 0=Yes; 1=No | **1** |
| Does scope of practice include well-woman care? 0 = No; 1 = Yes | **1** |
|  |  |
| **Medications - Authority of Midwife to Obtain and Administer Medications** |  |
| Do CNMs experience any challenges accessing any of the listed medications they are authorized to obtain and administer? 1= No; 0=Yes; 3= I do not know; 4=Not applicable | **1** |
| What level of prescription-writing authority do CNMs have? =4, Comprehensive list of medications given (specialized care medications - may require training, e.g. GBS prophylactic antibiotics, Pitocin)? = 3; 2= Limited list of medications allowed (routine and emergency medications, e.g. newborn antibiotic eye ointment, anti-hemorrhagic drugs), 1=Allowed only by physician written agreement, 0= Prohibited or not authorized | **4** |
| **Regulatory Board/Council/Advisory Committee** |  |
| Is midwifery representation on Board/Council/Advisory group required? 0=No; 1=APRN mentioned; CNM not specified; 2=Yes | **2** |
| **CERTIFIED MIDWIVES (CM)** |  |
| **Regulation & Medicaid** |  |
| Are CMs regulated? 1=No; 4=Yes | **4** |
| Is CM credential sufficient for licensure (additional steps for licensure may be required: TB test,state application requirements, state examination, etc.)? 0 = No; 1 = Yes | **1** |
| Is Medicaid reimbursement available? 0 = No; 1 = Yes, but challenges with reimbursement including birth site;3= Yes | **3** |
| **Autonomous Practice & Risk Assessment** |  |
| Is consultation/referral required (instead of recommended) by law for certain conditions? 0 = Unregulated state; 1=Required but difficult to access when needed; 2=Not required but difficult to access when initiated by CM for home and birth center; 3= Not required and easily accessed when initiated by CM | **3** |
| Is physician supervision required? 0=Yes; 1=No | **1** |
| Is a consultation agreement/collaborative practice agreement with a physician required? 0=Yes, formal written agreement/or formal consultation req'd/or unregulated; 1=Yes,but informal & unwritten; 3=No agreement req'd | **3** |
| **Scope of Practice** |  |
| Limited by law to childbearing year? 0 =Yes ; 1 = No | **1** |
| Scope of practice includes well-woman care? 0 = No; 1 = Yes | **1** |
| **Medications - Authority of Midwife to Obtain and Administer Medications** |  |
| What level of prescription-writing authority do CMs have? =4, Comprehensive list of medications given (specialized care medications - may require training, e.g. GBS prophylactic antibiotics, Pitocin)? = 3; 2= Limited list of medications allowed (routine and emergency medications, e.g. newborn antibiotic eye ointment, anti-hemorrhagic drugs), 1=Allowed only by physician prescription, 0= Prohibited or not authorized | **4** |
| **Regulatory Board/Council/Advisory Committee** |  |
| Is midwifery representation on Board/Council/Advisory group required? 0 = No; 1 = CM not specified; 2=Yes | **2** |
|  | **100** |
