# Supplementary material for: Mapping integration of midwives across the United States: Impact on access, equity, and outcomes
Source: PLoS One. 2018 Feb 21;13(2):e0192523. doi: 10.1371/journal.pone.0192523 (PMC5821332; doi:10.1371/journal.pone.0192523)
Supplement: S2 Table — (DOCX) [file pone.0192523.s002.docx]

**S2 Table. Association between MISS scores and state-level, race-specific neonatal death rates**

| Neonatal mortality rate^1^ | N (#states reporting)^2^ | Integration scores |
| --- | --- | --- |
| **Total** | 50 | -0.545** |
| Hispanic | 28 | -0.520** |
| Non-Hispanic white | 46 | -0.480** |
| Non-Hispanic black | 34 | -0.318* |

**Correlation is significant at the 0.01 level (2-tailed). *Correlation is significant at the 0.05 level (2-tailed).

Since a two-tailed test showed a significant association between total neonatal mortality and MISS scores we used a one-tailed test for subsequent analysis of race-specific correlations.

^1^ babies that died within 27 days of birth in the year 2013

^2^ Rates based on <20 deaths were excluded as they did not meet standards of statistical precision.
