# Supplementary material for: Mapping integration of midwives across the United States: Impact on access, equity, and outcomes
Source: PLoS One. 2018 Feb 21;13(2):e0192523. doi: 10.1371/journal.pone.0192523 (PMC5821332; doi:10.1371/journal.pone.0192523)
Supplement: S3 Table — (DOCX) [file pone.0192523.s003.docx]

**S3 Table. Correlations between licensure and birth outcomes/interventions**

|  | CM licensure (yes/no) | CMP licensure (yes/no) | MISS Scores |  |
| --- | --- | --- | --- | --- |
| Spontaneous Vaginal Birth | -0.116 | 0.254 | 0.402** |  |
| Vaginal birth after Cesarean | 0.059 | 0.146 | 0.330* |  |
| Induction | -0.018 | -0.240 | -0.275 |  |
| Preterm birth | -0.069 | -0.275 | -0.480** |  |
| Low birth weight | -0.016 | -0.124 | -0.353* |  |
| Cesarean section | 0.148 | -0.125 | -0.278* |  |
| Total neonatal death rate | -0.081 | -0.245 | -0.545** |  |
| Breastfeeding at birth | -0.109 | 0.433** | 0.584** |  |
| Breastfeeding - 6 months | -0.132 | 0.364** | 0.378** |  |

**Correlation is significant at the 0.01 level (2-tailed).*Correlation is significant at the 0.05 level (2-tailed).
