# Supplementary figures and images for: Mapping integration of midwives across the United States: Impact on access, equity, and outcomes
Source: PLoS One. 2018 Feb 21;13(2):e0192523. doi: 10.1371/journal.pone.0192523 (PMC5821332; doi:10.1371/journal.pone.0192523)

**S1 Fig. Scatter plot showing relationship between integration scores and neonatal death (2013)**


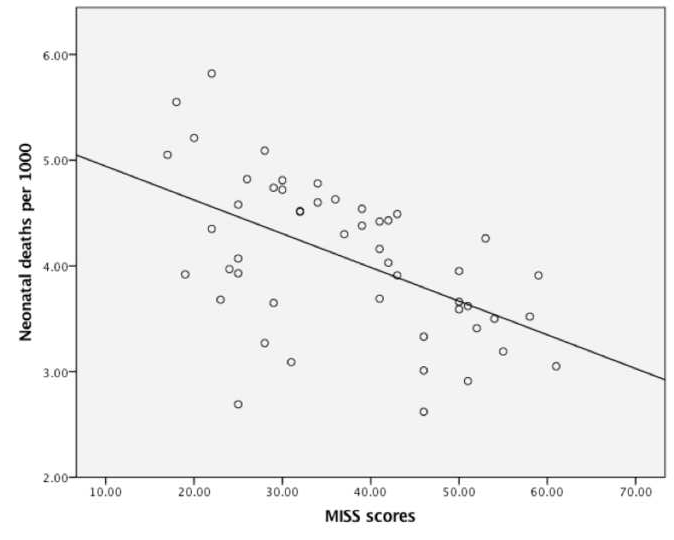

Supplement: S1 Fig — (DOCX) [file pone.0192523.s004.docx]
